# Quantifying the amount of physical rehabilitation received by individuals living with neurological conditions in the community: a scoping review

**DOI:** 10.1186/s12913-022-07754-4

**Published:** 2022-03-16

**Authors:** Tyler M. Saumur, Sarah Gregor, Yijun Xiong, Janelle Unger

**Affiliations:** 1 Rehabilitation Sciences Institute, University of Toronto, 160-500 University Ave, Toronto, ON M5G 1V7 Canada; 2 School of Physical Therapy, Western University, London, ON N6G 1H1 Canada

## Abstract

**Background:**

Physical rehabilitation is often prescribed immediately following a neurological event or a neurological diagnosis. However, many individuals require physical rehabilitation after hospital discharge. The purpose of this scoping review was to determine the amount of physical rehabilitation that individuals living in the community with neurological conditions receive to understand current global practices and assess gaps in research and service use.

**Methods:**

This scoping review included observational studies that 1) involved adults living with a neurological condition, and 2) quantified the amount of rehabilitation being received in the community or outpatient hospital setting. Only literature published in English was considered. MEDLINE, EMBASE, AMED, CINAHL, Cochrane Library, and PEDro databases were searched from inception. Two independent reviewers screened titles and abstracts, followed by full texts, and data extraction. Mean annual hours of rehabilitation was estimated based on the amount of rehabilitation reported in the included studies.

**Results:**

Overall, 18 studies were included after screen 14,698 articles. The estimated mean annual hours of rehabilitation varied greatly (4.9 to 155.1 h), with individuals with spinal cord injury and stroke receiving the greatest number of hours. Participants typically received more physical therapy than occupational therapy (difference range: 1 to 22 h/year). Lastly, only one study included individuals with progressive neurological conditions, highlighting a research gap.

**Discussion:**

The amount of rehabilitation received by individuals with neurological conditions living in the community varies greatly. With such a wide range of time spent in rehabilitation, it is likely that the amount of rehabilitation being received by most individuals in the community is insufficient to improve function and quality of life. Future work should identify the barriers to accessing rehabilitation resources in the community and how much rehabilitation is needed to observe functional improvements.

**Supplementary Information:**

The online version contains supplementary material available at 10.1186/s12913-022-07754-4.

## Background

Physical rehabilitation, such as physical and occupation therapy, is commonly prescribed following the diagnosis of a neurological condition or the occurrence of a neurological event. The goal of physical rehabilitation is to optimize physical functioning so people can continue to complete tasks that are important to them, as independently and safety as possible. More specifically, physical therapy typically views movement on a continuum while considering the physical, pathological, social, and psychological aspects [1]. Common therapeutic activities include transfer and gait training, strength exercises, and balance training [2]. Conversely, occupational therapy focuses on improving independence with activities of daily living using an approach that incorporates both physical and mental health [3]. The practice of occupational therapy involves tasks such as prescribing adaptive equipment, optimizing activities of daily living, and practicing fine motor tasks. Common tasks performed in occupational therapy may include problem solving, reaching to grasp a cup, and minimizing stimulation in public spaces. Despite approximately 0.5–2 h per day being spent on physical rehabilitation during inpatient rehabilitation [4–6], persistent physical impairments in neurological populations are often present long-term such as spasticity, pain, muscle weakness, and fatigue [7–9]. Accordingly, many individuals require ongoing physical rehabilitation after discharge from inpatient rehabilitation hospitals.

Quality of life – which may be one of the most important self-perceived measure of function – has been shown to improve with access to physical rehabilitation in the community setting [10]. The community setting can be operationalized as visiting an outpatient clinic, a community centre, or being visited in-home by a healthcare practitioner. Outpatient clinic use is more common than receiving services in-home [11]. Betterment in many of these functional domains can impact quality of life [12, 13], therefore highlighting the benefits of physical rehabilitation for individuals living with neurological conditions in the community. Outpatient programs have been shown to results in a manifold of improvements to functional independence, balance, and mobility [14–16].

Individuals with long-term neurological conditions have qualitatively reported that their physical rehabilitative needs are not met [17]. However, to our knowledge, no review has evaluated how much time is spent in physical rehabilitation following chronic neurological impairment, to better understand this gap in care quantitatively. It is important to understand current global practices to first gauge the current norms in the field of neurological rehabilitation. By assessing this data, we can then work towards determining the optimal time needed to improve physical function and provide recommendations for insurance companies or hospitals and direct future research studies and programs. Therefore, the purpose of this scoping review was to identify and describe studies that characterize the amount of outpatient or community physical rehabilitation received by those living with neurological conditions. The goal of this work is to inform future guidelines for community rehabilitation and provide a baseline amount of therapy for interventions targeting these populations.

## Methods

### Search strategy

This scoping review was conducted according to the guidelines presented in the PRISMA Extension for Scoping Reviews [18]. A protocol paper has been previously submitted for the present scoping review (Saumur et al., submitted). In brief, following a search of review registries to determine no similar review is currently in progress, an initial search strategy was developed in MEDLINE (Additional file 1) with the assistance of a research librarian surrounding the concepts of ‘Rehabilitation’, ‘Neurological Populations’, and ‘Time Factors’. The search strategy was then translated for application in Ovid Embase, Ovid Allied and Complementary Medicine (AMED), EBSCO Cumulative Index to Nursing and Allied Health Literature (CINAHL), and Scopus. The searches for journal articles in all databases were conducted on December 17^th^, 2020.

### Study selection & screening

Articles were uploaded to Covidence (Covidence, Victoria, Australia) where they were initially deduplicated. Articles were then screened for eligibility. Articles were included if they met the following criteria: 1) adults 18 years of age or older; 2) living in the community with a neurological condition defined as traumatic brain injury, multiple sclerosis, spinal cord injury, stroke, and Parkinson’s disease [19, 20]; 3) used an observational study design, 4) reported the amount of rehabilitation received; 5) published in English; and 6) have abstract and full text available in a peer-reviewed journal. Experimental studies, systematic/scoping reviews, and gray literature such as newspaper articles, reports, and dissertations were not included.

Two reviewers independently reviewed all deduplicated titles and abstracts to determine which articles to include for full text screening. If consensus could not be achieved between the two reviewers, the other team members were consulted to determine the article’s eligibility. During full text screening, two reviewers independently assessed the potential articles for their eligibility. If the article was not included, a reason was provided based on the inclusion criteria. The same procedures as title and abstract screening were used in the event of reviewer disagreement. Once full texts were selected for study inclusion, backwards citation tracking was conducted to consult the references of included studies for additional articles.

### Data extraction & analysis

Following full text screening, data extraction was performed based on a standardized, piloted extraction form developed by the research team. Data extracted included: year and country published in; study design and objectives; population and patient demographics included in the article; method of data collection; time spent in rehabilitation; and type of rehabilitation. The two primary reviewers independently extracted the data for each study and an additional team member cross-referenced the two data extraction forms with the included journal article. The collected data were then summarized using descriptive statistics (mean and standard deviation or median and interquartile range, accordingly) and presented in tabular and graphical forms. Annual time spent in rehabilitation was estimated in hours for each study based on the information provided. One hour was allocated for each session in the event that number of sessions was reported based on previous research, which has shown that 1 session is approximately 1h on average [21].

## Results

### Included studies

**Fig. 1:**
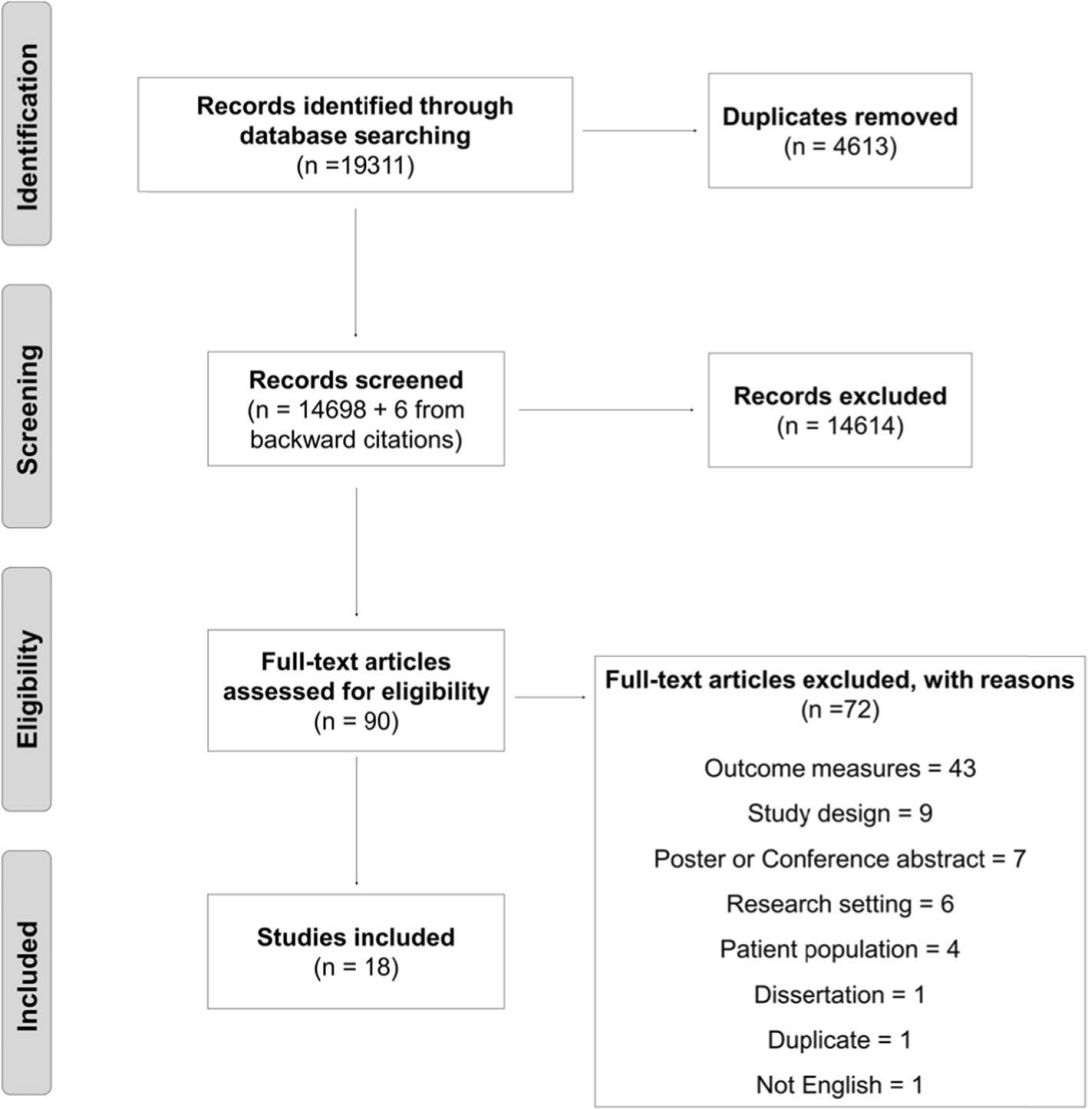
PRISMA diagram

A PRISMA diagram outlining the screening process can be found in Fig. 1. In brief, following deduplication, 14,698 articles were initially assessed for eligibility. Following title and abstract screening, 88 articles were selected for full text review. Of these 88 articles, 18 were included in this review. The main reasons for articles being excluded were due to the outcomes reported (58.6%, e.g., no mention of time spent in rehabilitation); study design (12.9%, e.g., experimental design); and poster or conference abstract (10.0%).

### Study Demographics

**Fig. 2:**
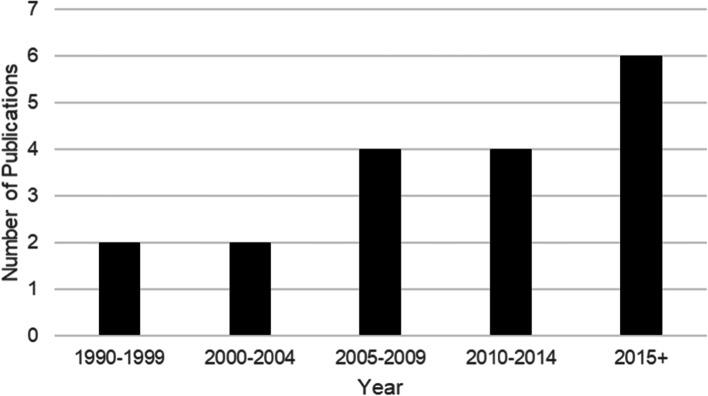
Year studies were published

Table 1 outlines the main objectives, study design, and participant demographics of the included studies. Most studies were conducted in the United States (*n* = 6) or the United Kingdom (*n* = 4). No research was found prior to 1990 in the literature, with an incremental increase in the presence of these studies over the last three decades (Fig. 2).

**Table 1:** General study information sorted alphabetically

**Citation**	**Objective**	**Design**	**Data Collection Tool**	**Population**	**Chronicity**
Backus et al. (2013) [39]	To examine the types and amounts of services provided to study participants during the first year after SCI, including during inpatient rehabilitation and post-discharge and the degree to which these services are associated with functional outcomes, social integration, and quality of life 1 year after SCI	Prospective, observational cohort, longitudinal, multi-centre	Phone interview	Spinal Cord Injury	Up to one year post injury
Freburger et al. (2018) [25]	To determine whether receipt of therapy and number and timing of therapy visits decreased hospital readmission risk in stroke survivors discharged home	Retrospective, cohort analysis	Health Claims data	Stroke	30 days post-hospital discharge
Freburger et al. (2018) [24]	To identify predictors of therapist use (any use, continuity of care, timing of care) in the acute care hospital and community (home or outpatient) for patients discharged home after stroke	Retrospective, cohort analysis	Health Claims data & hospital databases	Stroke	30 days post-hospital discharge
Geddes et al. (2001) [40]	To describe and compare six community services providing coordinated, multidisciplinary rehabilitation to people with stroke	Prospective, descriptive study	Collected by a team based on a designed protocol	Stroke	From 1.7 to 25.3 weeks post-stroke
Gladman et al. (1991) [23]	To survey the routine provision of PT and OT in outpatient and day hospitals in Nottingham	Longitudinal study, cross-sectional survey, and observational study	Records from outpatient departments and day hospitals, survey of PTs and OTs, observer during Rx	Stroke	From 1 to 61 days post-inpatient discharge
Green et al. (1999) [2]	To a survey the demand for physiotherapy for longer-term stroke-related mobility problems within the context of an established and comprehensive, community physiotherapy service	Cross-sectional	Survey	Stroke	> 1 year post-stroke with mobility problems
Grimley et al. (2020) [41]	The aims of this study were to describe current patterns and dose of rehabilitation received in various service configurations and settings in Queensland, Australia, over the first six months after stroke and to examine whether setting of rehabilitation is asso- ciated with differences in functional improvement	Prospective, observational cohort, multi-site	Medical Records	Stroke	Until 6 months post-stroke
Hodgkinson et al. (2000) [38]	To document service utilization by people with traumatic brain injury at different times postinjury and to identify factors that predict service use	Cross-sectional	Questionnaire	Traumatic Brain Injury	6 months to 17 years
Homaifar et al. (2009) [22]	To describe healthcare utilization and cost for veterans with TBI 4 to 40 years postinjury, taking into account age, years since injury, and severity of TBI	Cross-sectional	Electronic patient records	Traumatic Brain Injury	4 to 40 years (Years since injury: Total sample: 18.4 (15.0) ± 12.2; mild: 19.7 (17.0) ± 10.9; mod/severe: 18.0 (13.0) ± 12.7)
Huang et al. (2009) [42]	To investigate the impact of both timing and dose of rehabilitation delivery on the functional recovery of stroke patients	Retrospective cohort	Medical chart review	Stroke	From 1 to 12 months post-stroke
Jackson et al. (2014) [26]	To determine the cost of different components of these services (rehab, social support, equipment provision) during the first and second six-month period following discharge	Prospective longitudinal cohort	Questions with follow-up phone interview	Long-term neurological condition (stroke, TBI, ABI, SCI most common)	At 6 and 12 months post-discharge
Jalayondeja et al. (2011) [43]	To evaluate a model for community participation by Thai stroke victims 6 months post-stroke	Prospective longitudinal cohort	Prospective medical charting	Stroke	From 1 to 6 months post-stroke
Minet et al. (2020) [44]	To investigate the predictive value of disease-related factors, contextual factors, and functioning on the use of healthcare for 1 year after stroke	Prospective	Electronic patient records, structured interviews, and assessments	Stroke	From 3 to 12 months post-stroke
Ngo et al (2009) [45]	To examine use of physical therapy (PT) and occupational therapy (OT) among Medicare beneficiaries nationwide before and after the 1997 Balanced Budget Act (BBA), which introduced prospective payment for rehabilitation services	Retrospective observational	Health claims data	Stroke	Not reported
Pucciarelli et al. (2018) [46]	To describe the type and the amount of formal and informal care received by stroke survivors during the first year after home discharge and to identify the baseline predictors of the formal and informal care needs of stroke survivors	Prospective longitudinal observational	Questionnaire administered during interview	Stroke	From 3 to 12 months post-hospital discharge
Rhoda et al. (2009) [47]	To determine the Structure and Process of the rehabilitation of stroke patients at Community Health Centres (CHCs) in the Western Cape, South Africa	Cross-sectional	Patient registers and therapists’ records	Stroke	Up to 6 months post-stroke
Thompson et al. (2019) [48]	To determine the current state of stroke rehabilitation practice within the Wellington Community Older Adults, Rehabilitation and Allied Health (WCORA) team	Prospective cohort	Therapist tracking data form	Stroke	The first 4 weeks and first 3 months post-hospital discharge
Whiteneck et al. (2011) [49]	Examine amount and type of therapy services received in inpatient and post-discharge settings in the first year after spinal cord injury	Prospective observational longitudinal cohort	Patient interviews	Spinal Cord Injury	Up to one year post injury

The most commonly used data collection tools to evaluate the amount of time in physical rehabilitation were questionnaires, interviews, and surveys (*n* = 6); medical charts or electronic patient record (*n* = 5); health claims data (*n* = 3). Some studies used a variety of methods to gather this information (*n* = 2), or custom data collection sheets (*n* = 2) were also implemented in some studies.

Stroke was by far the most common condition studied (*n* = 13), with SCI (*n* = 2), TBI (*n* = 2), and multiple long-term neurological conditions (*n* = 1) also being evaluated. Chronicity of the condition varied from immediately following hospital discharge until 40 years post-injury. Half of the studies distinguished between occupational and physical therapy (*n* = 9), and the rest of the studies grouped all types of physical rehabilitation (*n* = 9).

### Time spent in rehabilitation

**Fig. 3:**
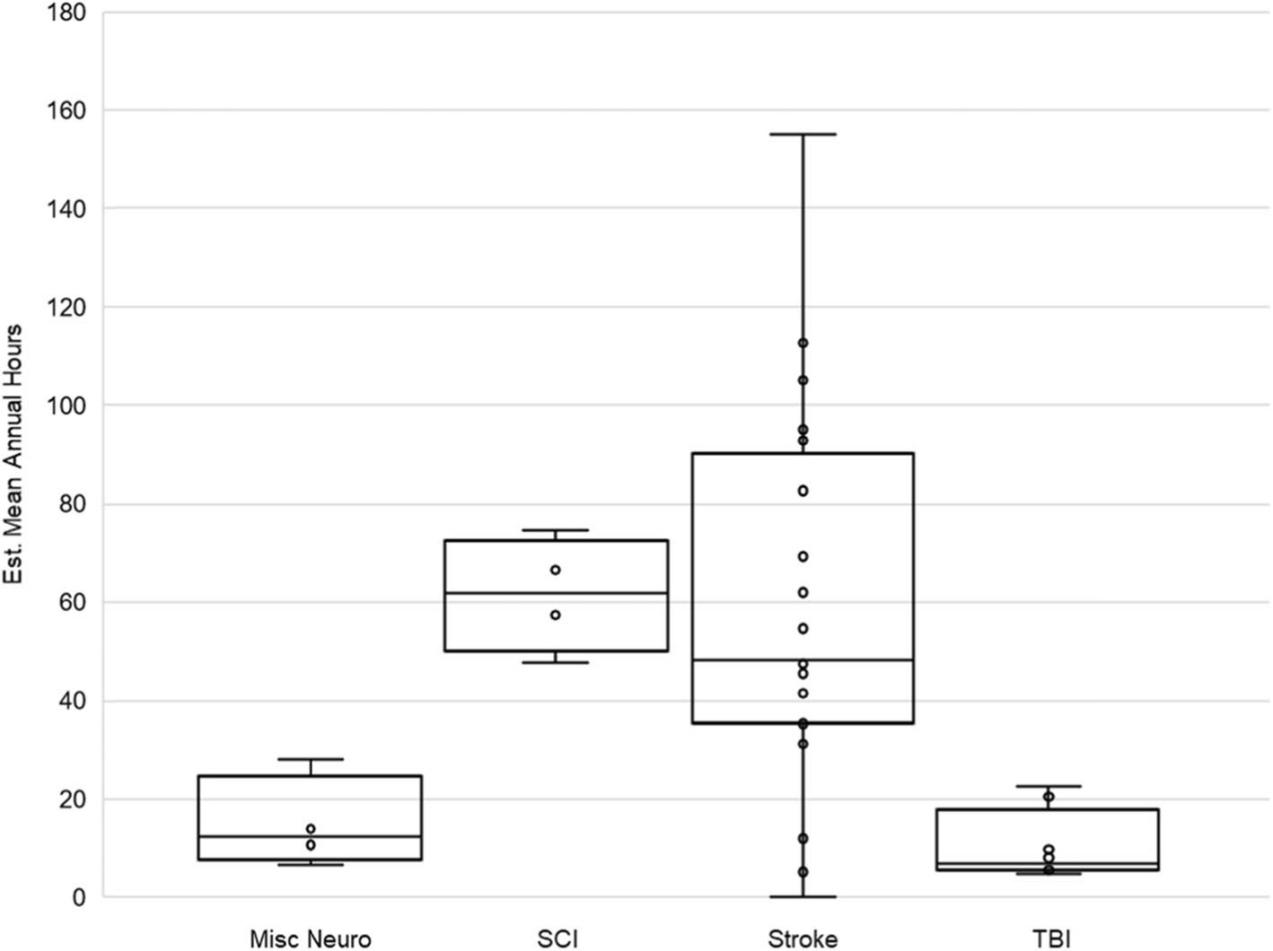
Estimated mean annual hours reported for each physical rehabilitation service, separated by neurological condition. Misc Neuro included stroke, traumatic brain injury, other acquired brain injury, spinal cord injury, peripheral neuropathy, and progressive long-term neurological conditions [26]

**Fig. 4:**
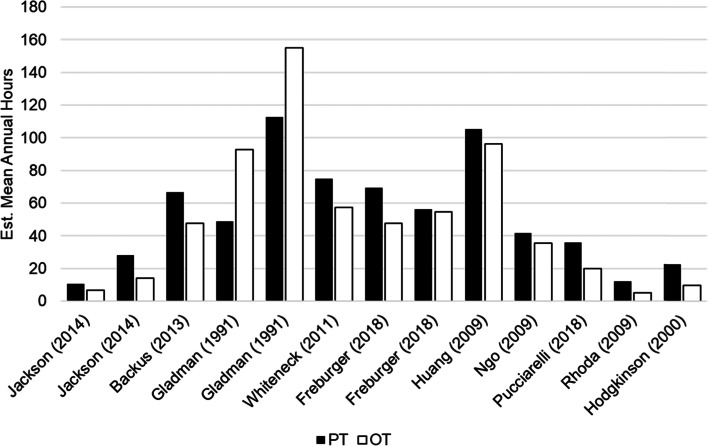
Estimated mean annual hours separated by service type. PT = physical therapy; OT = occupational therapy. Note: some studies compared service use in multiple settings (e.g., in-home and outpatient) and thus have been reported twice [23, 25, 26]

The estimated mean annual hours spent in rehabilitation ranged from 4.9 h in mild TBI [22] to 155.1 h in chronic stroke [23] (Table 2). On average, SCI and stroke received the most hours of rehabilitation (Fig. 3). In general, participants received more physical therapy than occupational therapy, regardless of condition (9 of 10 studies; Fig. 4). Regarding location of services, two of four studies found that patients utilized more in-home services compared to outpatient services [24, 25], whereas two studies found that more time was spent in outpatient services [23, 26].

**Table 2:** Annual time spent in rehabilitation, organized by population

**Citation**	**Country**	**Annual time spent in rehabilitation**	**Population**
Jackson et al. (2014) [26]	UK	In-home: PT – 10.7 h; OT – 6.6 h Outpatient: PT – 28.2 h; OT – 13.9 h	Long-term neurological condition (stroke, TBI, ABI, SCI most common)
Backus et al. (2013) [39]	USA	Outpatient/day program: PT – 66.5 ± 74 h; OT – 47.7 ± 59.1 h	Spinal Cord Injury
Whiteneck et al. (2011) [49]	USA	Outpatient/day program: PT – 74.7 ± 72.1 h; OT – 57.4 ± 66.2 h	Spinal Cord Injury
Freburger et al. (2018) [25]	USA	In-home: 82.7 h Outpatient: 62.0 h	Stroke
Freburger et al. (2018) [24]	USA	In-home: PT – 69.3 h; OT – 47.4 h Outpatient: PT– 56.0; OT – 54.7 h	Stroke
Geddes et al. (2001) [40]	UK	In-home: 31.2 ± 25.6 h	Stroke
Gladman et al. (1991) [23]	UK	Day hospital: PT – 48.5 h, OT – 92.7 h Outpatient: PT – 112.7 h, OT – 155.1 h	Stroke
Green et al. (1999) [2]	UK	In-home/rehabilitation centre: 45.5 h	Stroke
Grimley et al. (2020) [41]	AUS	Community rehabilitation: 12 h*	Stroke
Huang et al. (2009) [42]	CHN	Outpatient: PT – 105.0 ± 26.3 h; OT – 96.2 ± 26.0 h	Stroke
Jalayondeja et al. (2011) [43]	THA	Rehabilitation: 48 h	Stroke
Minet et al. (2020) [44]	SWE	Outpatient: 95 h	Stroke
Ngo et al (2009) [45]	USA	PT – 41.5 ± 6.8 h; OT – 35.3 ± 7.9 h	Stroke
Pucciarelli et al. (2018) [46]	ITA	PT – 35.7 h; OT – 20 h	Stroke
Rhoda et al. (2009) [47]	ZAF	Community rehabilitation: PT – 12.2 h; OT – 5.2 h	Stroke
Thompson et al. (2019) [48]	NZL	Community rehabilitation: 36.6 h	Stroke
Hodgkinson et al. (2000) [38]	AUS	Rehabilitation: PT – 22.7 h; OT – 9.8 h	Traumatic Brain Injury
Homaifar et al. (2009) [22]	USA	Outpatient: 10 years post-injury – 4.9 h (mild), 5.8 h (moderate/severe); 20 years post-injury – 8.1 h (mild), 5.7 h (moderate/severe); 30 years post-injury – 13.0 h (mild), 5.7 h (moderate/severe); 40 years post-injury – 20.6 h (mild), 5.6 h (moderate/severe)	Traumatic Brain Injury

## Discussion

The purpose of this study was to identify and describe studies that characterize the amount of outpatient or community physical rehabilitation received by those living with neurological conditions. We found that the amount of physical rehabilitation received by individuals with neurological conditions living in the community varied greatly. Those with spinal cord injury and stroke received a greater number of hours on average regardless of service and the majority of studies were conducted within the first year following injury. In general, however, more time was spent in physical therapy than occupational therapy.

The first main finding in this study was that individuals who experienced a spinal cord injury or stroke received a greater number of rehabilitation hours while living in the community compared to other populations. Reasons why these populations would receive more rehabilitation than other populations, such as traumatic brain injury are unclear; however, it does not appear to be linked with severity of injury as Homaifer and colleagues (2009) reported that the amount of rehabilitation received by those with mild traumatic brain injury was greater than those with moderate/severe injury beyond 10 years post-injury [22]. These findings may suggest that severity of impairment is not a key factor in service use for those living in the community; however, studies that directly compare functional severity with service use are needed.

A second main finding of this review was that the vast majority of studies reported increased hours spent in physical therapy compared to occupational therapy (ranging between 1 and 22 annual hours difference in those studies which reported greater physical therapy use). There are a few potential reasons for the finding. Firstly, there are approximately 37% more registered physical therapists than occupational therapists [27]. The availability of staff and resources likely explains this difference in service utilization in those with neurological impairments. In addition, there is some overlap in the roles of occupational and physical therapists [28], despite these roles being functionally quite distinct. For example, both professions may use strengthening exercises to improve physical function of the hand; however, the goal to implement these exercises may different (i.e., physical therapy – improve hand strength so can hold onto a walker; occupational therapy – improve hand strength so can dress oneself) [29]. However, many individuals may not know this difference between roles, and individuals in the community may be inclined to receive whichever service is readily available to them, regardless of the goal. It is important that individuals understand the difference between these types of physical rehabilitation so that their appropriate needs can be met. Indeed, therapists have reported factors such as lack of professional role clarity and restricted multidisciplinary team working as key barriers to providing community-based rehabilitation [30]. Taken together, this research highlights the importance of role clarity, accessibility, and perceived value as key factors influencing the amount of rehabilitation individuals participate in in the community.

With respect to the location of services, results were split with half of the studies finding patients utilized more in-home services compared to outpatient services [24, 25], whereas half found that more time was spent in outpatient services [23, 26]. This contrasts work by Godwin et al., (2011) which has shown that time spent in outpatient rehabilitation is more than three times that of in-home services. It is worth noting however, that the aforementioned study calculated rehabilitation utilization based on healthcare costs which likely impacted the findings. Furthermore, the utilization of resources available in the community likely differs by region and depends on features related to access such as distance to the nearest hospital or the equipment required. Therefore, while the location of rehabilitation used in the community may vary, a one-size-all approach should not be used when comparing patient needs and the resources available.

Despite our findings demonstrating that people with neurological diagnoses commonly do receive some physical rehabilitation after discharge from hospital inpatient units, the amount of rehabilitation received is likely insufficient. Research indicates many people continue to have disability long-term following diagnosis, and evidence shows that failure to access occupational and physical therapy is associated with continued issues following a brain injury [31, 32]. Furthermore, the evidence is clear that ongoing rehabilitation services do help long term [15]. However, patients frequently discuss short-term barriers to community rehabilitation such as problems ambulating and the inconvenience of attending sessions as well as long-term barriers related to finances and lack of interest or perceived need [33]. We also acknowledge that presently, access to these resources is limited. For example, in the United States, fewer than 10% of individuals living in the community with a stroke access occupational and physical therapy [34]. Thus, understanding the barriers to accessing the resources and how much rehabilitation is needed to observe functional improvements is critical.

### Where do we go from here?

The present scoping review highlighted various gaps in the literature pertaining to physical rehabilitation use in those with neurological conditions living in the community. Firstly, while we sought out to study individuals with discrete and progressive neurological conditions, we did not identify any studies on those with a degenerative condition with the exception of Jackson et al., (2014) which included various neurological conditions including 21 subjects (5%) with progressive long-term neurological conditions. It is important to note that the focus of rehabilitation differs between these two disease types. Rehabilitation for degenerative neurological disorders aims to manage the condition and slow the decline of physical function by developing compensatory strategies and increasing support over time [35]. Conversely, after a traumatic or ischemic event, rehabilitation focuses on reducing disability following the acute event to pre-injury functional capacities [36, 37]. Since treatment goals may differ between these groups, access to resources and tracking of these populations likely also differs. This can be appreciated by the lack of research pertaining to those with degenerative neurological conditions living in the community.

Furthermore, while studies did not track service use over time within the same cohort of subjects, it will be important for researchers to track service use longitudinally in order to further understand these trends and factors that affect them. As most of the studies included in this review focused on the first year following injury, there remains a large gap in services utilized over the long term in these populations. Determining the optimal timing and length of treatment is important in both progressive and non-progressive conditions as individuals have long-term needs related to improving or maintaining independence and slowing the rate of functional decline [35–37]. In the present study, the wide range of annual time spent in rehabilitation even within the same population, setting, and country, points to a lack of standardization and evidence-based practice in terms of the amount of rehabilitation received once discharged from the hospital. A goal of future research should be to determine the optimal amount of time needed to maximize the physical benefits of rehabilitation so that standards can be established. Notably, this determination of optimal usage of services can only be established through linking service use to functional outcomes. However, these findings provide the foundation for future development of clinical practice guidelines and can inform policies surrounding rehabilitation services in outpatient or community settings.

### Limitations

This review is not without its limitations. Firstly, the calculation of time spent in rehabilitation was estimated based on the number of sessions when time in minutes or hours was not provided. Many factors may influence the length of therapy sessions, and these may vary over time. However, the purpose of these calculations was to provide a broad picture of the landscape of community rehabilitation for those with neurological impairments. In addition, due to the small number of studies and only three studies including subjects more than one year following injury [2, 22, 38], disease chronicity was not considered which likely impacts rehabilitation time. Furthermore, articles were limited to those published in English and therefore we may have missed some studies published in different languages from other countries that could have been useful in gathering a more global perspective of rehabilitation use. This review also did not include experimental research, which may limit understanding of physical rehabilitation service programs that are in development, however the aim was to describe utilization of current services. Several included articles used data collection tools such as surveys and interviews, which rely on self-reporting and may be subject to recall bias. In addition, there was a lack of details regarding the variables reported within the included studies such as: number of therapists, health care delivery model, funding type, and accessibility; these features would greatly strengthen our ability to discuss the physical rehabilitation context of the included studies. Lastly, while this review sought to explore time spent in rehabilitation in both progressive and non-progressive neurological disease, there was limited research available, particularly in progressive groups. The treatment and access to resources likely differs between these patient groups and warrants further research.

## Conclusions

This scoping review found that the amount of physical rehabilitation received by individuals with neurological conditions living in the community varied greatly, with individuals who experienced a spinal cord injury or stroke receiving the greatest amount of care. In addition, more time is spent on average in physical therapy compared to occupational therapy in the community. These findings highlight the heterogeneity of physical rehabilitation received by individuals with a neurological condition and point to various avenues for future research including studying service use over time, the impact of community rehabilitation on functional outcomes and quality of life, and rehabilitation use in individuals with progressive neurological conditions.

## Supplementary Information

**Additional file 1:** Medline (OVID) Search Strategy.

## Data Availability

Not applicable.
