# Supplementary material for: Quantifying the amount of physical rehabilitation received by individuals living with neurological conditions in the community: a scoping review
Source: BMC Health Serv Res. 2022 Mar 16;22:349. doi: 10.1186/s12913-022-07754-4 (PMC8925183; doi:10.1186/s12913-022-07754-4)
Supplement: Supplementary file 1 — Additional file 1: Medline (OVID) Search Strategy. [file 12913_2022_7754_MOESM1_ESM.docx]

Appendix 1. Medline (OVID) Search Strategy

| **Line #** | **Search Terms** |
| --- | --- |
| 1 | exp Physical Therapy Modalities/ |
| 2 | exercise/ or physical conditioning, human/ or circuit-based exercise/ or endurance training/ or high-intensity interval training/ or plyometric exercise/ or resistance training/ |
| 3 | "physical and rehabilitation medicine"/ or rehabilitation/ |
| 4 | "activities of daily living"/ or animal assisted therapy/ or equine-assisted therapy/ or cardiac rehabilitation/ or dance therapy/ or early ambulation/ or exercise therapy/ or motion therapy, continuous passive/ or muscle stretching exercises/ or music therapy/ or neurological rehabilitation/ or stroke rehabilitation/ or occupational therapy/ or recreation therapy/ or rehabilitation, vocational/ or telerehabilitation/ |
| 5 | occupational therapy/ or physical therapy specialty/ |
| 6 | exp Community Participation/ |
| 7 | occupational health services/ |
| 8 | Retention in care/ or transitional care/ |
| 9 | rh.fs. |
| 10 | rehab*.tw,kf. |
| 11 | physical therap*.tw,kf. |
| 12 | physiotherap*.tw,kf. |
| 13 | occupational therap*.tw,kf. |
| 14 | ((exercis* or physical activit*) adj2 (technique* or therap* or program*)).tw,kf. |
| 15 | ((rehab* or physical therap* or physiotherap* or occupational therap*) adj2 (communit* or outpatient* or home care)).tw,kf |
| 16 | 1 or 2 or 3 or 4 or 5 or 6 or 7 or 8 or 9 or 10 or 11 or 12 or 13 or 14 or 15 |
| 17 | Time Factors/ |
| 18 | (time or hour* or minute*).ti. |
| 19 | ((time or hour* or minute*) adj2 spent).tw,kf. |
| 20 | (time adj2 "use").tw,kf. |
| 21 | 17 or 18 or 19 or 20 |
| 22 | exp Brain Injuries/ |
| 23 | exp Stroke/ |
| 24 | exp Parkinsonian Disorders/ |
| 25 | exp Multiple Sclerosis/ |
| 26 | exp Spinal Cord Diseases/ |
| 27 | (neuro* adj (injur* or trauma* or disease* or disorder* or condition*)).tw,kf. |
| 28 | ((brain or head) adj (injur* or trauma* or disease* or disorder* or condition*)).tw,kf. |
| 29 | stroke.tw,kf. |
| 30 | cerebrovascular accident.tw,kf. |
| 31 | (parkinson* adj (disease* or disorder*)).tw,kf. |
| 32 | multiple sclerosis.tw,kf. |
| 33 | ((spinal cord* or spin*) adj (injur* or trauma* or disease* or disorder* or condition*)).tw,kf. |
| 34 | 22 or 23 or 24 or 25 or 26 or 27 or 28 or 29 or 30 or 31 or 32 or 33 |
| 35 | 16 and 21 and 34 |
| 36 | Limit 35 to English language |
